# Comparison of a Potential Hospital Quality Metric With Existing Metrics for Surgical Quality–Associated Readmission

**DOI:** 10.1001/jamanetworkopen.2019.1313

**Published:** 2019-04-19

**Authors:** Laura A. Graham, Hillary J. Mull, Todd H. Wagner, Melanie S. Morris, Amy K. Rosen, Joshua S. Richman, Jeffery Whittle, Edith Burns, Laurel A. Copeland, Kamal M. F. Itani, Mary T. Hawn

**Affiliations:** 1 Health Services Research and Development Unit, Birmingham VA Medical Center, Birmingham, Alabama; 2 Department of Surgery, University of Alabama at Birmingham, Birmingham; 3 Center for Healthcare Organization and Implementation Research, Boston VA Healthcare System, Boston, Massachusetts; 4 Department of Surgery, Boston University School of Medicine, Boston, Massachusetts; 5 Veterans Affairs, Palo Alto, Veterans Affairs Medical Center, Palo Alto, California; 6 Department of Surgery, Stanford University School of Medicine, Palo Alto, California; 7 Milwaukee Veterans Affairs Medical Center, Milwaukee, Wisconsin; 8 Department of Surgery, Medical College of Wisconsin, Milwaukee; 9 Veterans Affairs Central Western Massachusetts Healthcare System, Leeds; 10 University of Massachusetts Medical School, Worcester; 11 Harvard University School of Medicine, Boston, Massachusetts

## Abstract

**Importance:**

The existing readmission quality metric does not meaningfully distinguish readmissions associated with surgical quality from those that are not associated with surgical quality and thus may not reflect the quality of surgical care.

**Objective:**

To compare a quality metric that classifies readmissions associated with surgical quality with the existing metric of any unplanned readmission in a surgical population.

**Design, Setting, and Participants:**

Cohort study using US nationwide administrative data collected on 4 high-volume surgical procedures performed at 103 Veterans Affairs hospitals from October 1, 2007, through September 30, 2014. Data analysis was conducted from October 1, 2017, to January 24, 2019.

**Main Outcomes and Measures:**

Hospital-level rates of unplanned readmission (existing metric) and surgical readmissions associated with surgical quality (new metric) in the 30 days following hospital discharge for an inpatient surgical procedure.

**Results:**

The study population included 109 258 patients who underwent surgery at 103 hospitals. Patients were majority male (94.1%) and white (78.2%) with a mean (SD) age of 64.0 (10.0) years at the time of surgery. After case-mix adjustment, 30-day surgical readmissions ranged from 4.6% (95% CI, 4.5%-4.8%) among knee arthroplasties to 11.1% (95% CI, 10.9%-11.3%) among colorectal resections. The new surgical readmission metric was significantly correlated with facility-level postdischarge complications for all procedures, with ρ coefficients ranging from 0.33 (95% CI, 0.13-0.51) for cholecystectomy to 0.52 (95% CI, 0.38-0.68) for colorectal resection. Correlations between postdischarge complications and the new surgical readmission metric were higher than correlations between complications and the existing readmission metric for all procedures examined (knee arthroplasty: 0.50 vs 0.48; hip replacement: 0.44 vs 0.18; colorectal resection: 0.52 vs 0.42; and cholecystectomy: 0.33 vs 0.10). When compared with using the existing readmission metric, using the new surgical readmission metric could change hip replacement–associated payment penalty determinations in 28.4% of hospitals and knee arthroplasty–associated penalties in 26.0% of hospitals.

**Conclusions and Relevance:**

In this study, surgical quality–associated readmissions were more correlated with postdischarge complications at a higher rate than were unplanned readmissions. Thus, a metric based on such readmissions may be a better measure of surgical care quality. This work provides an important step in the development of future value-based payments and promotes evidence-based quality metrics targeting the quality of surgical care.

## Key Points

**Question:**

Does the current unplanned readmission quality metric reflect the quality of surgical care among veterans undergoing 4 high-volume inpatient surgical procedures?

**Findings:**

In this cohort study of 109 258 patients who underwent surgery at 103 US Veterans Affairs hospitals, a measure of surgical quality–associated readmission was more highly correlated than the unplanned readmission metric with postdischarge complication in all procedures assessed. Redefining the current readmission metric to assess surgical quality would change knee arthroplasty–associated penalties in 26.0% of hospitals and hip replacement–associated penalties in 28.4% of hospitals.

**Meaning:**

Although existing metrics may be effective measures of surgical quality for some procedures, there is still a need for a more refined measure to accurately assess the quality of surgical care in other procedures.

## Teaser

This cohort study analyzes data from US Veterans Affairs hospitals to compare the existing metric of any unplanned readmission, which may not reflect the quality of surgical care, with a metric that classifies readmissions as likely associated with surgical quality in high-volume inpatient surgical procedures.

## Introduction

In 2012, the Hospital Readmissions Reduction Program (HRRP) of the Centers for Medicare & Medicaid Services (CMS) targeted admissions for 3 medical conditions: acute myocardial infarction, heart failure, and pneumonia. Payment penalties were imposed on hospitals with high risk-adjusted readmission rates. In 2014, CMS excluded planned readmissions from the program, and in 2015, added 2 surgical procedures—total hip replacement and total knee arthroplasty—to the targeted index admissions.^1^ No changes were made to the definition of an unplanned readmission. The current metrics include all unplanned readmissions following a surgical procedure and ignore the quality of surgical care. With the addition of 2 new surgical readmission measures, hospital executives called on their surgical staff to reduce readmissions associated with surgery. Current research has shown that surgical readmissions are not fully explained by factors available from the hospital discharge summary.^2,3,4,5,6,7,8^ Since the elimination of planned readmissions from the readmission quality metric, no effort has been made, to our knowledge, to improve the relationship between unplanned readmissions and surgical quality.

Some of our group developed a potential definition of a surgical quality–associated readmission in a prior study using a modified Delphi process.^8,9^ In the current study, we operationalize and compare the new surgical quality–associated readmission metric to the unplanned readmission metric that is currently in use. We assess the correlation of both with postdischarge complication rates, a measure that is widely believed to reflect surgical quality. We hypothesize that the new surgery quality–associated readmission metric will be more highly correlated than the existing readmission metric with postdischarge complication rates. Support for our hypothesis would provide evidence that the new quality metric is more representative of the quality of surgical care. As a secondary objective, we examine how the new metric would change hospital payment penalties as assessed by HRRP if it were used in lieu of the current readmission metric.

## Methods

This study uses a prospective study design and administratively collected data from a national cohort of patients. This article adheres to the current Strengthening the Reporting of Observational Studies in Epidemiology (STROBE) reporting guideline for cohort studies. The study protocol was reviewed and approved by the Veterans Affairs Central Institutional Review Board, which also waived the need to obtain informed consent.

### Setting and Participants

The study population included patients who underwent surgery at 103 Veterans Health Administration (VHA) hospitals between October 1, 2007, and September 30, 2014. Surgical procedures were identified from the Veterans Administration Surgical Quality Improvement Program (VASQIP). The VASQIP captures approximately 83% of all eligible major noncardiac surgical procedures.^10^ Only the 4 most common inpatient surgical procedures were included. These procedures were (1) knee arthroplasty, (2) total and partial hip replacement, (3) colorectal resection, and (4) cholecystectomy or common bile duct exploration. Patients with a length of stay less than 2 days and patients who died in the hospital were excluded from this analysis.

### Defining Readmission Quality Metrics

Readmissions were defined as any inpatient admission to a VHA hospital in the 30 days following hospital discharge from the index surgical admission. Inpatient admissions were queried from the VHA Corporate Data Warehouse inpatient domain. Planned readmissions were excluded using the current CMS coding algorithm.^1^ The final unplanned readmission quality metric was defined as any unplanned readmission to a VHA hospital in the 30 days following hospital discharge from the index surgical admission.

Surgical quality–associated readmissions (surgical readmissions) were identified by *International Classification of Diseases, Ninth Revision* (*ICD-9*) codes using a definition reached by consensus of a panel of experts on readmission.^8,9^ Prior to this analysis, a modified Delphi process was used to assess whether a subsequent 30-day readmission was associated with the quality of a surgical procedure occurring during the index hospitalization. Reasons for readmission were determined by a review of the primary and secondary *ICD-9* codes. Panelists were presented with the top 50 reasons for readmission, representing 90% of all reasons for readmission in the sample. Panelists were asked, “Does the readmission reason reflect possible surgical quality of care problems in the index admission?” Readmissions were considered surgical readmissions if the panel agreed that they were “likely related” or “directly related to index quality.” After 3 rounds, the expert panel identified 16 reasons for readmission that were associated with the quality of the index surgical procedure (eTable 1 in the Supplement). These reasons included wound-associated readmission, hemorrhage or hematoma, ostomy complication, and other infectious outcomes.^8,9^ After further review by the study team (all authors), readmissions associated with all device complications and device malfunctions were also included in the definition of surgical readmissions for this analysis. The top 10 readmission reasons that were not classified as associated with surgery are shown in eTable 2 in the Supplement.

### Additional Variables

Postdischarge complications were used as an indicator of surgical quality for this analysis. Complications were defined as any VASQIP nurse–identified postoperative complication. Since VASQIP only collects postoperative complications up to 30 days after the operation date, a time frame of 14 days postdischarge was chosen to minimize the effect of right censoring among patients with longer postoperative stays.

Patient age, sex, race, functional status, American Society of Anesthesiologist physical status classification, history of acute myocardial infarction, history of peripheral vascular disease, recent diagnosis of depression, and current diabetes were included in the case-mix adjustment to account for variation across patient populations. Operative time, work relative value unit (RVU), and emergent surgery status were also included in the case-mix adjustment to account for variations within procedure type. Work RVU is a measure of surgical procedure complexity defined by CMS for each specific procedure code. When determined by VASQIP, this measure represents the highest work RVU *Current Procedural Terminology* code coded for the procedure, not just the work RVU for the principal *Current Procedural Terminology* code.

In addition to the VASQIP-assessed comorbidities and surgery characteristics, the hospital-level excess readmission ratio for each facility was calculated using the CMS HRRP formulas for fiscal year 2014.^11^ The excess readmission ratio is used to determine CMS payment penalties due to excess readmission rates. This was calculated as the case-mix adjusted predicted readmissions divided by the case-mix adjusted expected readmissions. The excess readmission ratio was calculated for both the unplanned readmission metric and the surgical readmission metric. Facilities with excess readmission ratios less than 1.00 were assumed to have no CMS payment penalty. Facilities with excess readmission ratios between 1.00 and 1.03 were assumed to have CMS payment penalties equivalent to the excess readmission ratio (eg, 1.021 = 2.1%). Penalties were capped at 3% for an excess readmission ratio of 1.03 or greater. The potential change in penalties experienced by each facility was determined by comparing the estimated penalty for the hospital when the current unplanned readmission metric was used and the estimated penalty for the hospital when the surgical-quality readmission metric was used.

### Statistical Analysis

All analyses were stratified by surgical procedure type because surgical procedures vary in indication, predictors, and outcomes. All patient and operative characteristics were aggregated to the facility level for each procedure. Years during which facilities had fewer than 25 inpatient surgical procedures were excluded from the analysis, consistent with CMS methods for assessing hospital quality.^4^ Unadjusted outcomes were calculated as aggregated hospital-level rates of readmission, surgical readmission, and postdischarge complications for each procedure. Case-mix adjusted outcomes were then determined using mixed-effects logistic regression with random intercepts for the hospitals and fixed effects for patient and operative characteristics.

Spearman rank correlation coefficients (ρ) were used to examine associations among the case-mix adjusted metrics. Bootstrapping with 1000 repetitions was used to calculate 95% CIs for the Spearman rank correlation coefficients. Two-sided tests were used throughout all analyses, and an α level of .05 determined statistical significance. Data management was conducted in SAS, version 9.4 (SAS Institute Inc). All statistical analyses and plots were constructed in R, version 3.4.1 (The R Foundation). Mixed models were output using the glmer function in the lme4 package, and plots were constructed using the ggplot2 package.^12,13^ Data analyses were conducted from October 1, 2017, to January 24, 2019.

## Results

The final analytic sample consisted of 103 VHA hospitals accruing patients across a 7-year study period. Thirty-one hospitals were excluded because of low procedure volume across all study years (ie, <25 cases per year). The study population included 109 258 patients who underwent inpatient surgery at these 103 hospitals. Most patients were male (94.1%), white (78.2%), and older (mean [SD] age, 64.0 [10.0] years) individuals. The most prevalent procedures occurred in the orthopedic and general surgery specialties: knee arthroplasty, hip replacement, colorectal resection, and inpatient cholecystectomy. Orthopedic procedures were most prevalent across the 7-year study period with means (SDs) of 477.4 (237.0) knee arthroplasties per hospital and 255.2 (112.1) hip replacements per hospital. There was a mean (SD) of 234.4 (95.9) colorectal resections per hospital and a mean (SD) of 146.0 (66.5) inpatient cholecystectomy procedures per hospital (Table 1).

**Table 1.** Characteristics of 109 258 Patients Undergoing the 4 Most Common Inpatient Surgical Procedures

Characteristic	Patients, %				
	Overall	Knee Arthroplasty (n = 45 833)	Hip Replacement, Total and Partial (n = 24 338)	Colorectal Resection (n = 15 514)	Cholecystectomy/Common Bile Duct Exploration (n = 15 514)
Overall					
No. of facilities performing procedure	103	96	95	103	103
No. of procedures per facility, mean (SD)	1060.8 (443.1)	477.4 (237.0)	255.2 (112.1)	234.4 (95.9)	146.0 (66.5)
Patient characteristics					
Age, mean (SD), y	64.0 (10.0)	64.0 (9.0)	63.9 (10.6)	65.0 (10.8)	62.5 (13.2)
Female sex	5.9	6.3	5.0	4.0	8.8
Black race/ethnicity (n = 104 849)	16.0	14.7	19.4	16.8	12.9
Partially or totally dependent at surgery (n = 109 907)	5.1	2.1	8.8	5.9	7.4
ASA classification >3 at surgery (n = 109 915)	6.3	2.6	5.7	11.4	10.0
Patient comorbidities					
Recent MI history	0.22	0.06	0.14	0.50	0.40
PVD history	1.5	0.9	1.3	2.3	2.5
Depression diagnosis within 1 y (n = 109 694)	16.9	18.5	16.7	13.1	18.3
Diabetes	23.0	23.3	17.5	24.3	28.7
Operative characteristics					
Operative time, mean (SD), h	2.4 (1.1)	2.2 (0.7)	2.3 (0.9)	3.2 (1.6)	2.0 (1.0)
Work RVU, mean (SD)	21.9 (4.6)	22.7 (2.0)	21.8 (2.5)	25.5 (4.3)	13.7 (3.4)
Emergent surgery (n = 109 924)	0.4	0.2	1.7	9.1	13.0

Abbreviations: ASA, American Society of Anesthesiologists; MI, myocardial infarction; PVD, peripheral vascular disease; RVU, relative value unit.

As shown in Table 1, patient characteristics, comorbidities, and operative characteristics varied not only by procedure type but also within specialty (orthopedic vs general). Patients who underwent colorectal resection were older and had longer operative times and higher work RVUs. By contrast, those who underwent inpatient cholecystectomy were younger with lower work RVU procedures but were more commonly identified as emergent procedures (13.0%) within VASQIP. General surgery procedures (colorectal resection and cholecystectomy) were more likely to be performed on sicker patients as assessed by American Society of Anesthesiologist classification (classification >3: colorectal resection 11.4% and cholecystectomy 10.0% vs knee arthroplasty 2.6% and hip replacement 5.7%).

### Overlap of Readmission Metrics With Postdischarge Complication Rates

Although the number of readmissions categorized as surgical quality–associated was always smaller than the number categorized as unplanned, this difference varied by procedure, with small differences for knee arthroplasty (4.6% [95% CI, 4.5%-4.8%] vs 5.0% [95% CI, 4.9%-5.2%]), hip replacement (5.3% [95% CI, 5.2%-5.5%] vs 5.4% [95% CI, 5.3%-5.5%]), and colorectal resection (11.1% [95% CI, 10.9%-11.3%] vs 13.1% [95% CI, 12.8%-13.4%]) but larger differences for cholecystectomy procedures (6.0% [95% CI, 5.9%-6.0%] vs 9.7% [95% CI, 9.6%-9.7%]) (Table 2). There was substantial overlap between the two definitions. More than two-thirds of unplanned readmissions (71.6%) following surgery were also categorized as surgical quality–associated by our definition, whereas 84.5% of surgical quality–associated readmissions were also categorized as unplanned.

**Table 2.** Variation in Outcomes by Surgical Procedure Type Across 103 Facilities

Facilities Outcome	Mean (95% CI), %	
	Unadjusted	Adjusted^a^
Knee arthroplasty		
30-d Surgery-associated readmission	4.7 (4.3-5.1)	4.6 (4.5-4.8)
30-d Any unplanned readmission	5.1 (4.7-5.5)	5.0 (4.9-5.2)
14-d Postoperative complication	1.8 (1.6-2.0)	1.7 (1.7-1.8)
Hip replacement, total and partial		
30-d Surgery-associated readmission	5.6 (5.0-6.1)	5.3 (5.2-5.5)
30-d Any unplanned readmission	5.7 (5.2-6.2)	5.4 (5.3-5.5)
14-d Postoperative complication	2.5 (2.1-2.8)	2.2 (2.1-2.3)
Colorectal resection		
30-d Surgery-associated readmission	10.7 (10.0-11.4)	11.1 (10.9-11.3)
30-d Any unplanned readmission	12.8 (12.0-13.5)	13.1 (12.8-13.4)
14-d Postoperative complication	7.9 (7.2-8.6)	8.1 (7.7-8.5)
Cholecystectomy and common bile duct exploration		
30-d Surgery-associated readmission	5.9 (5.4-6.4)	6.0 (5.9-6.0)
30-d Any unplanned readmission	9.7 (9.1-10.2)	9.7 (9.6-9.7)
14-d Postoperative complication	3.6 (3.2-3.9)	3.6 (3.5-3.6)

^a^ Case-mix adjusted outcomes were determined using mixed-effects logistic regression models with random intercepts for the hospitals, and with patient and operative characteristics as the independent variables. Models were adjusted for patient age, sex, race/ethnicity, functional status, American Society of Anesthesiologist classification, history of acute myocardial infarction, history of peripheral vascular disease, recent diagnosis of depression, current diabetes diagnosis, operative time, work relative value unit, and emergent surgery status.

The overall postdischarge complication rate was 3.7%. Most postdischarge complications identified by VASQIP were wound associated (29.6% superficial infections, 12.4% wound dehiscence, 8.1% deep wound infection, and 9.2% organ/space surgical site infections). Postdischarge complication rates varied widely across procedure types, from a mean (95% CI) of 1.8% (1.6%-2.0%) for knee arthroplasty to 7.9% (7.2%-8.6%) for colorectal resection (Table 2). Case-mix adjustment decreased the within-procedure variation as shown by the smaller confidence intervals, but variation across surgical procedure types remained, highlighting the importance of stratified analyses.

**Figure 1.**
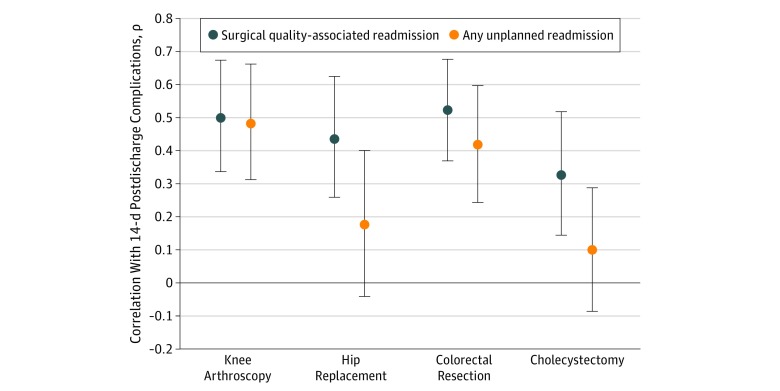
Magnitude of Correlation Between Readmission Metrics and 14-Day Postdischarge Complications by Procedure Type Data points represent mean; error bars, 95% CIs.

Figure 1 displays the overlap between the readmission metrics and postdischarge complication rates after case-mix adjustment. Overall, both the unplanned and surgical readmission metrics were correlated with 14-day postdischarge complication rates (surgical: ρ = 0.40; 95% CI, 0.23-0.56; unplanned: ρ = 0.35; 95% CI, 0.17-0.53). The new surgical readmission metric was significantly correlated with 14-day postdischarge complication rates for all procedures, with ρ coefficients ranging from 0.33 (95% CI, 0.13-0.51) for cholecystectomy to 0.52 (95% CI, 0.38-0.68) for colorectal resection. The correlation between the surgical-quality readmission metric and 14-day postdischarge complication rates was consistently higher than the correlation between the current unplanned readmission metric and 14-day postdischarge complication rates for all types of procedures (knee arthroplasty: 0.50 vs 0.48; hip replacement: 0.44 vs 0.18; colorectal resection: 0.52 vs 0.42; and cholecystectomy: 0.33 vs 0.10). This was particularly notable for hip replacement procedures and cholecystectomy procedures, in which 14-day postdischarge complication rates were not significantly associated with the current unplanned readmission metric (hip replacement: ρ = 0.18; 95% CI, −0.03 to 0.40; *P* = .08 and cholecystectomy: ρ = 0.10; 95% CI, −0.09 to 0.29; *P* = .33) (Figure 1).

We also observed significant correlations between both readmission metrics for knee arthroplasty (ρ = 0.77; 95% CI, 0.69-0.87) and colorectal resection (ρ = 0.82; 95% CI, 0.75-0.90) and a significant correlation between these metrics for hip replacement (ρ = 0.39; 95% CI, 0.22-0.58). For cholecystectomy procedures, there was no significant correlation between the two readmission metrics (ρ = 0.17; 95% CI, −0.04 to 0.39; *P* = .09). For every procedure, postdischarge complications were significantly correlated with surgical-quality readmission rates (Figure 1).

### Changes to Hospital Payment Penalties

**Figure 2.**
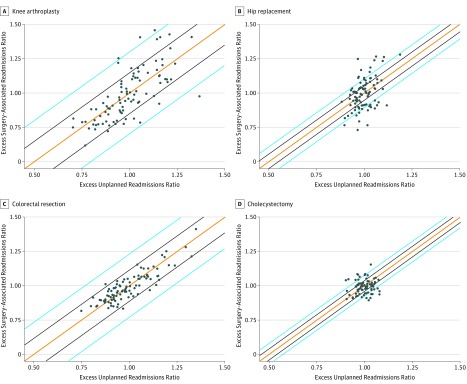
Scatterplots for Adjusted Facility-Level Observed/Expected Readmission Ratios Orange line indicates perfect correlation; black lines, agreement to within 1 SD; blue lines, agreement to within 2 SDs.

Figure 2 displays scatterplots of the final HRRP excess readmission ratios for each readmission quality metric. These were examined to understand the distribution and variation of excess readmission rates across all hospitals. There was large variation across hospitals in the ratio of excess unplanned readmissions for both knee arthroplasty and colorectal resection. In addition, few facilities had excess surgical readmission ratios more than 2 SDs outside the excess unplanned readmission ratio distribution for these procedures (4.2% for knee arthroplasty and 0.0% for colorectal resection, Figure 2). By contrast, there was very little variation across facilities in excess unplanned readmissions for hip replacement procedures but large variation in the excess surgical readmission ratio. The estimated excess readmission ratios for hip replacement procedures differed by more than 2 SDs in 28.4% of facilities. Trends among cholecystectomy procedures were similar to those among hip replacement procedures except for smaller variation in excess surgical readmissions (Figure 2).

Payment penalties experienced by facilities varied by the readmission quality metric used (Table 3). Changes were more frequent for cholecystectomy procedures (65.0%) compared with knee arthroplasty procedures (26.0%), hip replacement procedures (28.4%), and colorectal resections (18.4%). Among cholecystectomy procedures, 30.1% of hospitals would see a decreased penalty if the surgical readmission metric were used in lieu of the current unplanned readmission metric, and 34.9% would see an increased or added penalty. Twenty facilities (39.1%) with no cholecystectomy-associated penalty under the current readmission metric would be penalized if the surgical readmission metric were used. Among the 52 facilities that would be penalized for excess unplanned readmission rates following cholecystectomy, 31 facilities (59.6%) would see a reduction in the penalty amount if the surgical readmission metric were used in lieu of the current unplanned readmission metric.

**Table 3.** Changes to Hospital Readmissions Reduction Program Penalties If the Surgical Quality–Associated Readmissions Metric Is Used in Lieu of the Current Unplanned Readmission Quality Metric

Surgical Procedure	No. (%) of Hospitals				
	No Change		Change to Penalty		
	No Penalty (n = 166)	Penalty (n = 93)	Increased (n = 25)	Decreased (n = 59)	Penalty Added (n = 54)
Knee arthroplasty (n = 96)	44 (45.8)	27 (28.1)	5 (5.2)	12 (12.5)	8 (8.3)
Hip replacement (n = 95)	34 (35.8)	34 (35.8)	0	8 (8.4)	19 (20.0)
Colorectal resection (n = 103)	57 (55.3)	27 (26.2)	4 (3.9)	8 (7.8)	7 (6.8)
Cholecystectomy (n = 103)	31 (30.1)	5 (4.9)	16 (15.5)	31 (30.1)	20 (19.4)

## Discussion

The current unplanned readmission metric used by the HRRP was developed for a medical population but is being applied to a surgical population. Although providing some indication of the overall quality of inpatient care, the existing unplanned readmission metric overestimates the contribution of readmissions to surgical quality. Our proposed surgical quality–associated readmission metric was correlated with postdischarge surgical complications in all procedures. The correlation was higher than the correlation between the current readmission metric and postdischarge complications for all procedures assessed. This finding indicates that our new surgical readmission metric may be a better measure of surgical quality than the current readmission metric. This study highlights some of the issues with using the existing readmission metric to gauge the quality of surgical care.

Consistent with prior research,^14,15^ we found variation in surgical readmissions across procedures types, within surgical specialties, and across hospitals, further stressing the importance of accurate case-mix adjustment and procedure-specific models. Our findings of an unplanned readmission rate of 5.0% for knee arthroscopy and 5.4% for hip replacement match the 2014 CMS HRRP–reported unplanned readmission rate for knee arthroscopy or hip replacement (5.3%).^16^ Although no studies to our knowledge have examined the overlap of the current unplanned readmission metric with surgical quality, a few studies have attempted to estimate the number of unplanned readmissions that are potentially associated with the index surgical procedure.^1,17,18,19,20,21^ We found that 71.6% of unplanned readmissions were associated with the index surgical procedure. Rosen and colleagues estimated a smaller number (ie, 42%) that were deemed “clinically related.”^17^ Another recent study in an English population found that only 53% of unplanned readmissions were “surgical readmissions” associated with the index hip replacement or knee arthroscopy.^22^ Compared with that for current research, our surgical readmission metric is more conservative, classifying more readmissions as surgical quality–associated.

This brings to light an important limitation of the current research: the lack of a consistent definition of surgical readmission. To accurately assess and improve the quality of surgical care, surgical quality metrics should be appropriately designed. There is little guidance on how to define a surgical quality–associated readmission.^23^ The previous Delphi panel identified nonquality readmission reasons, such as mental health diagnoses, cardiac arrhythmias, peripheral vascular disease, and hematuria.^9^ Despite this rigorous method, we still overestimate the proportion of surgical readmissions compared with other studies.^17,18,22^ Designing a surgical quality–associated readmission metric is necessary before additional surgical procedures are targeted by the CMS HRRP. Without a readmission metric tailored to surgical quality, we are only measuring the same outcome in different settings. This contributes to the increasing burden of quality metric assessment experienced by our health care professionals without shedding light on potential areas for improvement in services.^24^

The current definition of any unplanned readmission following surgery may also be contributing to our inability to accurately predict readmissions after surgery. As with all predictive models, outcome specification is extremely important. Consistent with the HRRP hospital quality metric, surgical readmission studies typically identify any unplanned readmission after discharge from surgery as a surgical readmission.^25^ This is not the case, as our results and other studies show. Before building accurate models to predict surgical readmissions, it is important that we correctly identify the subset of readmissions that are associated with the quality of care delivered during the index surgery (ie, “surgical quality–associated readmissions”). As our findings suggest, the effect of this definition may vary across surgical procedure types, necessitating procedure-specific methods.

### Limitations

As with all studies in the VHA, we are limited in generalizability outside of the veteran population owing to the unique population characteristics. Our definition of readmission is also limited to readmissions to VHA hospitals. We suspect that we are missing approximately 10% to 15% of readmissions occurring in this cohort when the patient is readmitted to a non-VHA hospital.^26^ It is also important to keep in mind that we have limited our sample of eligible surgical procedures to inpatient procedures with a length of stay greater than 2 days and in which the patient was discharged alive. Although cholecystectomy procedures and common bile duct explorations were the fourth most frequent procedure in our cohort, these procedures are often performed with a length of stay shorter than 2 days; thus, the results for cholecystectomies suggest that those performed in our sample likely represented a more complicated procedure or sicker patients than is typical of cholecystectomies.

Although not a limitation, it is important to keep in mind that our definition of surgical quality–associated readmission is the result of a consensus-building process and still requires further development.^9^ To ensure the most accurate definition, we convened a group of 14 medical and surgical professionals that our study team identified as experts in surgical readmissions. The resulting readmission reasons determined to be likely associated or directly associated with the quality of surgical care are provided in eTable 1 in the Supplement. The top 10 reasons for readmissions that were not associated with surgical quality in 4 procedure samples are presented in eTable 2 in the Supplement. Although we used rigorous qualitative methods to develop this definition, further work may refine it.

## Conclusions

Postdischarge complication rates were more highly correlated with the new surgical-quality readmission metric than with the current readmission metric for all procedures assessed. These findings suggest that the currently used readmission metric may not be an appropriate measure of the quality of surgical care for these procedures, two of which are already being assessed by the CMS HRRP. These findings have implications for the design, use, and interpretation of readmission-targeted quality metrics when assessing the quality of surgical care. They represent the next steps in appropriately measuring surgical quality in our mission to provide value-based care. Future research on surgical readmissions should focus on defining readmissions following discharge from surgery in association with the surgery or the episode of hospitalization associated with the surgery.

## Notes

Supplement. **eTable 1.** ICD-9 and ICD-10 Codes for Surgical Quality-Related Readmissions **eTable 2.** Top 10 Primary Diagnosis Codes for Non-Surgery Related Readmissions
