# Supplementary material for: Comparison of a Potential Hospital Quality Metric With Existing Metrics for Surgical Quality–Associated Readmission
Source: JAMA Netw Open. 2019 Apr 19;2(4):e191313. doi: 10.1001/jamanetworkopen.2019.1313 (PMC6481441; doi:10.1001/jamanetworkopen.2019.1313)
Supplement: Supplement. — eTable 1. ICD-9 and ICD-10 Codes for Surgical Quality-Related Readmissions eTable 2. Top 10 Primary Diagnosis Codes for Non-Surgery Related Readmissions [file jamanetwopen-2-e191313-s001.pdf]

Supplementary Online Content

Graham LA, Mull HJ, Wagner TH, et al. Comparison of a potential hospital quality metric with existing metrics for surgical quality–associated readmission. *JAMA Netw Open*. 2019;2(4):e191313. doi:10.1001/jamanetworkopen.2019.1313

eTable 1. ICD-9 and ICD-10 Codes for Surgical Quality-Related Readmissions

eTable 2. Top 10 Primary Diagnosis Codes for Non-Surgery Related Readmissions

This supplementary material has been provided by the authors to give readers additional information about their work.

© 2019 Graham LA et al. *JAMA Network Open*.

eTable 1. ICD-9 and ICD-10 Codes for Surgical Quality-Related Readmissions

Readmission Reasons	ICD-9	ICD-10
**Wound-Related Codes**		
Postoperative Abscess/ Septicemia	998.59	K6811, T814XXA
Acute Abscess/ Diffuse Cellulitis/ Lymphangitis	682.0, 682.1, 682.2, 682.3, 682.4, 682.5, 682.6, 682.7, 682.8, 682.9	K122, L03119, L03129, L03211, L03212, L03213, L03221, L03222, L03317, L03319, L03329, L03811, L03818, L03891, L03898, L0390, L0391
Wound Disruption/ Dehiscence/ Rupture	998.30, 998.31, 998.32, 998.33	T8130XA, T8131XA, T8132XA, T8133XA
Amputation Infection	997.62	T8740, T8741, T8742, T8743, T8744
Infected Postoperative Seroma	998.51	T814XXA, T792XXA
Peritoneal Abscess	567.22, 540.1	K651, K353
**Bleeding**		
Hemorrhage or Hematoma	285.1, 459.0, 958.2, 998.11, 998.12, 998.13	D62, R58, T792XXA, D7801, D7802, D7821, D7822, D7831, D7832, D7833, D7834, E3601, E3602, E89810, E89811, E89820, E89821, E89822, E89823, G9731, G9732, G9751, G9752, G9761, G9762, G9763, G9764, H59111, H59112, H59113, H59119, H59121, H59122, H59123, H59129, H59311, H59312, H59313, H59319, H59321, H59322, H59323, H59329, H59331, H59332, H59333, H59339, H59341, H59342, H59343, H59349, H59351, H59352, H59353, H59359, H59361, H59362, H59363, H59369, H9521, H9522, H9541, H9542, H9551, H9552, H9553, H9554, I97410, I97411, I97418, I9742, I97610, I97611, I97618, I97620, I97621, I97622, I97630, I97631, I97638, I97640, I97641, I97648, J9561, J9562, J95830, J95831, J95860, J95861, J95862, J95863, K9161, K9162, K91840, K91841, K91870, K91871, K91872, K91873, L7601, L7602, L7621, L7622, L7631, L7632, L7633, L7634, M96810, M96811, M96830, M96831, M96840, M96841, M96842, M96843, N9961, N9962, N99820, N99821, N99840, N99841, N99842, N99843, T888XXA
**Gastrointestinal-Related Codes**		

© 2019 Graham LA et al. *JAMA Network Open*.

Ostomy Care/ Complications	V55.0, V55.1, V55.2, V55.3, V55.4, V55.5, V55.6, 569.60, 569.61, 569.69	K9400, K9402, K9409, K9410, K9412, K9419, Z430, Z431, Z432, Z433, Z434, Z435, Z436
**Device Complications**		
Catheter-Related Bloodstream Infection	996.62, 996.69, 999.31, 999.32, 999.33	T80211A, T80212A, T80219A, T827XXA, T8579XA
Other Device-Related Complications	996.00, 996.01, 996.02, 996.03, 996.04, 996.09, 996.1, 996.2, 996.30, 996.31, 996.32, 996.39, 996.40, 996.41, 996.42, 996.43, 996.44, 996.45, 996.46, 996.47, 996.49, 996.51, 996.52, 996.53, 996.54, 996.55, 996.56, 996.57, 996.59, 996.60, 996.61, 996.63, 996.64, 996.65, 996.66, 996.67, 996.68, 996.70, 996.71, 996.72, 996.73, 996.74, 996.75, 996.76, 996.77, 996.78, 996.79	M979XXA, T8201XA, T8202XA, T8203XA, T8209XA, T82110A, T82111A, T82120A, T82121A, T82190A, T82191A, T82211A, T82212A, T82213A, T82218A, T82221A, T82222A, T82223A, T82228A, T82390A, T82391A, T82392A, T8249XA, T82518A, T82519A, T82528A, T82529A, T82538A, T82539A, T82590A, T82591A, T82593A, T82595A, T82598A, T82599A, T826XXA, T827XXA, T82817A, T82818A, T82827A, T82828A, T82837A, T82838A, T82847A, T82848A, T82855A, T82856A, T82857A, T82858A, T82867A, T82868A, T82897A, T82898A, T829XXA, T83090A, T83091A, T83092A, T83098A, T83193A, T83198A, T8329XA, T8339XA, T83498A, T83510A, T83511A, T83512A, T83518A, T83590A, T83591A, T83592A, T83593A, T83598A, T8361XA, T8362XA, T8369XA, T8381XA, T8382XA, T8383XA, T8384XA, T8385XA, T8386XA, T8389XA, T839XXA, T84019A, T84029A, T84039A, T84059A, T84069A, T84099A, T84119A, T84129A, T84199A, T84498A, T8450XA, T8460XA, T847XXA, T8481XA, T8482XA, T8483XA, T8484XA, T8485XA, T8486XA, T8489XA, T849XXA, T8509XA, T85190A, T85192A, T85193A, T85199A, T8529XA, T85398A, T8549XA, T85590A, T85691A, T85692A, T85693A, T85694A, T85698A, T8571XA, T85730A, T85731A, T85732A, T85733A, T85734A, T85735A, T85738A, T8579XA, T85810A, T85818A, T85820A, T85828A, T85830A, T85838A, T85840A, T85848A, T85850A, T85858A, T85860A, T85868A, T85890A, T85898A, T859XXA, T86820, T86821

© 2019 Graham LA et al. *JAMA Network Open*.

**Renal Failure and Electrolyte Disorders**		
Other Fluid and Electrolyte Disorders	276.0, 276.1, 276.2, 276.3, 276.4, 276.50, 276.51, 276.52, 276.61, 276.69, 276.7, 276.8, 276.9	E860, E861, E869, E870, E871, E872, E873, E874, E875, E876, E8770, E8771, E8779, E878
Acute Renal Failure	584.5, 584.6, 584.7, 584.8, 584.9	N170, N171, N172, N178, N179
**Other**		
Anemia	280.0, 280.1, 280.9, 285.1, 285.9	D1800, D1801, D1809, D62, D649
Urinary Tract Infection	599.0, 996.64	N390, T83510A, T83511A, T83512A, T83518A
Bacterial Pneumonia	482.0, 482.1, 482.2, 482.3, 482.30, 482.31, 482.32, 482.39, 482.4, 482.40, 482.41, 482.49, 482.8, 482.81, 482.82, 482.83, 482.84, 482.89, 482.9, 485, 486, 507.0, 514	A481, J14, J150, J151, J1520, J15211, J15212, J1529, J153, J154, J155, J156, J158, J159, J180, J182, J189, J690, J811
PSI Postoperative Sepsis	038.0, 038.10, 038.11, 038.19, 038.2, 038.3, 038.4, 038.41, 038.42, 038.43, 038.44, 038.49, 038.8, 038.9, 785.52, 785.59, 995.91, 995.92, 998.0	A400, A401, A403, A408, A409, A4101, A4102, A411, A412, A413, A414, A4150, A4151, A4152, A4153, A4159, A4181, A4189, A419, A5486, B377, R571, R6520, R6521, T8110XA, T8111XA, T8112XA
PSI Postoperative Pulmonary Embolism or Deep Vein Thrombosis	415.11, 415.19, 451.11, 451.19, 451.2, 451.81, 451.9, 453.40, 453.41, 453.42, 453.87, 453.89, 453.9	I2690, I2699, I8010, I80209, I80211, I80212, I80213 I80219, I80232, I80233, I80239, I80291, I80292, I80293, I80299, I803, I809, I82210, I82220, I82221, I82290, I82401, I82402, I82403, I82409, I82411, I82412, I82413, I82419, I82421, I82422, I82423, I82429, I82431, I82432, I82433, I82439, I82449, I82499, I824Y1 I824Y2, I824Y3, I824Y9, I824Z9, I82609, I82619, I82629, I82890, I8290, I8291, I82A19, I82B19, I82C19, T800XXA, T81718A, T8172XA, T82817A, T82818A

Note: PSI=Patient Safety Indicator, developed by the Agency for Healthcare Research and Quality. http://www.qualityindicators.ahrq.gov/Modules/psi_overview.aspx

ICD10 codes are from the 2018 General Equivalence Mappings. https://www.cms.gov/Medicare/Coding/ICD10/2018-ICD-10-CM-and-GEMs

© 2019 Graham LA et al. *JAMA Network Open*.

eTable 2. Top 10 Primary Diagnosis Codes for Non-Surgery Related Readmissions

**Knee Arthroplasty**	**%**	**Hip Replacement**	**%**
Specific ICD-9 Codes (Primary)		*Specific ICD-9 Codes (Primary)*	
338.18 –Acute Postoperative Pain	7%	719.45 –Joint Pain (Pelvis)	3%
718.56 –Ankylosis of the Joint (Lower Leg)	5%	491.21 –Acute Exacerbation of COPD	3%
719.46 –Joint Pain (Lower Leg)	4%	780.2 –Syncope and Collapse	3%
780.2 –Syncope and Collapse	2%	835.00 –Dislocation of Hip	3%
786.59 –Chest Pain	2%	780.60 –Fever	2%
719.16 –Hemarthrosis (Lower Leg)	2%	782.3 –Edema	2%
729.81 –Swelling of Limb	2%	338.18 –Acute Postoperative Pain	2%
719.56 –Joint Stiffness (Lower Leg)	2%	427.31 –Atrial Fibrillation	2%
995.1 –Angioneurotic Edema	2%	428.0 –Congestive Heart Failure	2%
427.89 –Cardiac Dysrhythmia	1%	729.81 –Swelling of Limb	2%
**Colorectal Resection**	**%**	**Cholecystectomy**	**%**
Specific ICD-9 Codes (Primary)		*Specific ICD-9 Codes (Primary)*	
560.9 –Intestinal Obstruction	12%	997.4 –Other Digestive System Complications	6%
997.4 –Other Digestive System Complications	9%	577.0 –Acute Pancreatitis	6%
997.49 –Other Digestive System Complications	7%	574.50 -Choledocholithiasis	5%
789.00 –Abdominal Pain	3%	997.49 –Other Digestive System Complications	4%
560.1 –Paralytic Ileus	3%	789.01 –Abdominal Pain	3%
564.00 –Unspecified Constipation	2%	338.18 –Acute Postoperative Pain	2%
008.45 –Clostridium Difficile	2%	574.51 –Calculus of Bile Duct with Obstruction	2%
578.1 –Melena	2%	576.1 –Cholangitis	2%
338.18 –Acute Postoperative Pain	2%	008.45 –Clostridium Difficile	2%
558.9 –Other Noninfectious Gastroenteritis and Colitis	2%	428.0 –Congestive Heart Failure	1%

© 2019 Graham LA et al. *JAMA Network Open*.
